# In Response

**DOI:** 10.4269/ajtmh.16-0314b

**Published:** 2016-08-03

**Authors:** Karine Sévère, Vanessa Rouzier, Stravinsky Benedict Anglade, Claudin Bertil, Patrice Joseph, Alexandra Deroncelay, Marie Marcelle Mabou, Peter F. Wright, Florence Duperval Guillaume, Jean William Pape

**Affiliations:** ^1^ Les Centres GHESKIO, Port-au-Prince, Haiti. E-mails: karinesevere@gheskio.org, vrouzier@gheskio.org, vegatch@hotmail.com, claudinbert1902@gmail.com, pjoseph@gheskio.org, and mmabou@gheskio.org; ^2^ Department of Pediatrics, Dartmouth Medical School, Hanover, New Hampshire. E-mail: peter.f.wright@dartmouth.edu; ^3^ Ministry of Public Health and Population, Port-au-Prince, Haiti; ^4^ Center for Global Health, Department of Medicine, Weill Cornell Medical College, New York, New York. E-mail: jwpape@gheskio.org

Dear Sir:

We read with interest the letter to the editor by Rebaudet and others “Questioning the Effectiveness of Oral Cholera Vaccine in Port-au-Prince Slums” promoting the simplistic and dogmatic approach to cholera control with only water, sanitation, and hygiene (WASH) programs.^1^ This is contradictory to their own admission that the oral cholera vaccine (OCV) is at least 57% effective and hence comparable to the 55% efficacy of the highly used typhoid vaccine.^2^ More recent trials indicate an OCV efficacy of 65–70% for up to 5 years^3^ with short-term efficacy of 85% at 6 months.^4^ Further recent data indicate that even a single dose of OCV provides 40% protection against all cholera and 63% against severely dehydrating episodes for at least 6 months.^5^ Although providing clean water and improved sanitation are the cornerstone of cholera eradication and other waterborne diseases control and there has been a major increase in donor funding for WASH projects since 2010, there is limited evidence of success of this intervention. Haiti's National Plan to eliminate cholera in Haiti within the next decade identifies $1.6 billion in need for WASH.^6^

In our study, OCV was introduced as a complementary measure after 2 years of well-described WASH activities in a cholera hotspot in urban slums located within 15 m of our cholera treatment center (CTC). The impressive 97.5% effectiveness of the combined WASH (primarily provision of chlorine at home) plus OCV is remarkable by any account. As this was not a case–control trial, the only comparison of vaccine efficacy possible is between individuals within the slum who received OCV and those from the same slum who were not vaccinated. Rebaudet and others noted that if the vaccinated and unvaccinated groups were perfectly matched, we would have expected the rate of non-cholera diarrhea to be similar between the two groups. However, there were significantly more cases of non-cholera dehydrating disease in the unvaccinated group (348/17,643) than in the vaccinated group (37/52,357) living in the same area. It is hard to explain this decrease in non-cholera diarrheal illness in the vaccinated group as due to improved sanitation that was uniquely adopted by the vaccinated group or major socioeconomic differences that could exist between these two groups living side by side. Noted is that the percent of enteric illness caused by cholera in the vaccinated group (32%) is much lower than in the unvaccinated group (60%).

As mentioned by Rebaudet and others and discussed in our article, vaccine recipients could have presented elsewhere for care except that the GHESKIO CTC was the closest and only CTC that remained operational 24/7 throughout the entire study period. Indeed, most of the other CTCs set up in 2010 had already closed by the end of 2012, or remained open intermittently. Even for simply practical reasons, patients with severe diarrhea would have sought care at the closest CTC. Moreover, our CTC is well known in that community where we have been providing free medical care for over 30 years, our in-hospital mortality rate is among the lowest in Haiti (0.3%), and we have hundreds of community health agents working in the five slums where we provided OCV to advise and direct potential patients to our center for medical evaluation.

Haiti's cholera outbreak remains one of the largest in the world, with 29,642 cases reported in 2015 alone.^7^,^8^ Why then would anyone truly interested in cholera control not promote the use of all available effective and complementary methods, namely both OCV and home water chlorination? Our objective has always been to provide the most effective interventions for the control and eradication of cholera in Haiti. In contrast to Rebaudet and others, we have a 34-year history of working in Haiti and developing evidence-based models of care that are scaled up nationally with the Ministry of Health for the benefit of the country. In 1979, we introduced oral rehydration salt leading to a marked decrease in national infantile mortality, and since 1982 developed and expanded integrated models for the prevention and treatment of human immunodeficiency virus/acquired immunodeficiency syndrome, sexually transmitted diseases, and tuberculosis.^9^ Facts are stubborn, and it is quite remarkable that since our combined interventions of home chlorination plus OCV, we have not had one single case of culture-confirmed cholera in our vaccinees since September 2013.

The time is ripe to scale-up the proven feasible and effective combination of OCV with home water chlorination at the national level: the World Health Organization (WHO) recommends the use of OCV, the vaccine stockpile is increasing with the availability of a fourth manufacturer to be approved under the WHO pre-qualification program (Janssen-Cilag, UK, Shantha Biotechnics, India; Eubiologics, South Korea; Incepta Vaccine Ltd., Bangladesh) and additional funding support of $115 million from 2014 to 2018 has been approved by the Global Alliance for Vaccine and Immunisation.^10^ There should be no competing interest for donor funding for such complementary and synergistic interventions. Although long-term definitive infrastructure support for sanitation and potable water are essential and should be the ultimate objective for complete cholera eradication, OCV plus home chlorination can be offered immediately at the national level to curb the unnecessary morbidity and mortality in the population. This is the best tool we have to help control cholera in Haiti now, and if successful could be a model for other countries as well.
